# RNase H1 directs origin-specific initiation of DNA replication in human mitochondria

**DOI:** 10.1371/journal.pgen.1007781

**Published:** 2019-01-03

**Authors:** Viktor Posse, Ali Al-Behadili, Jay P. Uhler, Anders R. Clausen, Aurelio Reyes, Massimo Zeviani, Maria Falkenberg, Claes M. Gustafsson

**Affiliations:** 1 Department of Medical Biochemistry and Cell Biology, University of Gothenburg, Gothenburg, Sweden; 2 MRC Mitochondrial Biology Unit, University of Cambridge, Cambridge, United Kingdom; The University of Western Australia, AUSTRALIA

## Abstract

Human mitochondrial DNA (mtDNA) replication is first initiated at the origin of H-strand replication. The initiation depends on RNA primers generated by transcription from an upstream promoter (LSP). Here we reconstitute this process in vitro using purified transcription and replication factors. The majority of all transcription events from LSP are prematurely terminated after ~120 nucleotides, forming stable R-loops. These nascent R-loops cannot directly prime mtDNA synthesis, but must first be processed by RNase H1 to generate 3′-ends that can be used by DNA polymerase γ to initiate DNA synthesis. Our findings are consistent with recent studies of a knockout mouse model, which demonstrated that RNase H1 is required for R-loop processing and mtDNA maintenance in vivo. Both R-loop formation and DNA replication initiation are stimulated by the mitochondrial single-stranded DNA binding protein. In an RNase H1 deficient patient cell line, the precise initiation of mtDNA replication is lost and DNA synthesis is initiated from multiple sites throughout the mitochondrial control region. In combination with previously published in vivo data, the findings presented here suggest a model, in which R-loop processing by RNase H1 directs origin-specific initiation of DNA replication in human mitochondria.

## Author summary

Human mitochondria contain a double-stranded DNA genome that codes for key components of the oxidative phosphorylation system. The mitochondrial DNA (mtDNA) is replicated by a replication machinery distinct from that operating in the nucleus and mutations affecting individual replication factors have been associated with an array of rare, human diseases. In the present work, we demonstrate that RNase H1 directs origin-specific initiation of DNA replication in human mitochondria and that disease-causing mutations may impair this process. A unique feature of mtDNA replication is that primers required for initiation of leading-strand DNA replication are produced by the mitochondrial transcription machinery. A substantial fraction of all transcription events is prematurely terminated about 120 nucleotides downstream of the promoter and the RNA remains firmly associated with the genome, forming R-loops. Interestingly, the free 3′-end of these R-loops cannot directly prime initiation of DNA synthesis, but must first be processed by RNase H1. The process is stimulated by the mitochondrial single-stranded DNA binding protein and faithfully reconstitutes replication events mapped in vivo. In combination with mapping of replication events in fibroblasts derived from patients with mutations in *RNASEH1*, our findings point to a possible model for replication initiation in human mitochondria similar to that previously described in the *E*. *coli* plasmid, ColE1.

## Introduction

Mitochondrial DNA (mtDNA) is a 16.6 kb circular, double-stranded DNA (dsDNA) molecule that contains genes for 13 components of the oxidative phosphorylation (OXPHOS) system as well as the 22 tRNAs and 2 rRNAs required for their translation. The two strands can be separated by CsCl gradient density centrifugation and are accordingly referred to as the heavy (H) and light (L) strands. Polycistronic transcription of the two strands is initiated from the L-strand and H-strand promoters (LSP and HSP, respectively) and carried out by a transcription machinery consisting of a single subunit RNA polymerase (POLRMT), and transcription factors A (TFAM) and B2 (TFB2M) [1, 2]. Transcription is further stimulated by the mitochondrial transcription elongation factor (TEFM), which forms a stable ternary complex with the elongating POLRMT and template DNA [3–6].

Mitochondrial DNA is synthesized by the trimeric DNA polymerase γ (POLγ) that consists of one catalytic subunit (POLγA) and two identical accessory subunits (POLγB), which act to increase processivity [7–10]. The replicative DNA helicase TWINKLE is a hexameric protein and its activity is stimulated by the mitochondrial single-stranded DNA binding protein (mtSSB) [11, 12]. According to the strand displacement model for mtDNA replication, mtDNA synthesis is initiated from two separate origins of replication, OriH and OriL, one for each strand. DNA synthesis first commences at OriH and proceeds in one direction to produce a nascent H-strand [1, 13]. When the replication machinery has synthesized approximately two thirds of the H-strand, it passes OriL, which is exposed in its single-stranded conformation and activated. OriL adopts a stem-loop structure and POLRMT initiates synthesis of short RNA primers from a poly-dT stretch in the loop region. These primers are used by POLγ to initiate L-strand synthesis with the parental H-strand as template [14, 15]. H-strand and L-strand DNA replication subsequently proceed until two complete daughter molecules have been formed and separated in a Topoisomerase 3α-dependent process [16].

In the literature, there is some confusion with regard to the exact localization of OriH [1]. It is often defined as a single position, 191 in human mtDNA, since prominent free 5′-ends on DNA have been identified at this site [17]. It should however be noted that RNA to DNA transitions have never been identified at this position. How these 5′-ends are actually formed is not understood, but they are potentially the result of extensive primer processing far beyond the location of the actual RNA to DNA transition sites. The processing involves MGME1 and disease-causing mutations affecting this enzyme lead to accumulation of replication intermediates with incomplete processing of 5′-ends [18].

According to the current model for replication initiation, transcription initiated at LSP provides RNA primers for initiation at OriH [19–21]. In support of this notion, loss of POLRMT in a knockout mouse model abolishes primer synthesis in vivo [22]. The switch between primer formation and full-length transcription takes place in a region immediately downstream of LSP containing three evolutionary conserved sequence blocks, CSBI-III, and RNA to DNA transitions in newly synthesized H-strands have been mapped to multiple sites surrounding these elements [19–21]. Newly transcribed RNA remains associated with the CSB-region, forming R-loops that are resistant to RNase A and RNase T1 treatment [23–25]. The unique stability of these R-loops is explained by a G-quadruplex structure, which is formed co-transcriptionally between nascent RNA and the non-template DNA strand at CSBII. This G-quadruplex structure also stimulates premature transcription termination downstream of CSBII [26–28]. The 3′-ends of the terminated transcripts roughly overlap with RNA to DNA transitions mapped in the CSBII-region [26, 29], which has led to the hypothesis that sequence-dependent transcription termination may be responsible for primer formation. There is however no published experimental evidence demonstrating that transcripts prematurely terminated at CSBII can be directly used to prime DNA synthesis. In addition, a molecular understanding of the primer formation and replication initiation reaction is still missing.

Recently, a knockout mouse model demonstrated that RNase H1 is required for R-loop degradation in vivo. In addition, depletion of RNase H1 caused a reduction in mtDNA levels, suggesting that the enzyme is required for mtDNA replication [30]. These in vivo findings raise the possibility that RNase H1 may play a role in R-loop processing and primer formation. RNase H1 is an RNase H enzyme capable of cleaving RNA-DNA hybrids. It can cleave hybrids that are down to approximately 6 nucleotides in length [31]. The enzyme can also cleave Okazaki fragment-like structures, leaving approximately two ribonucleotides next to the RNA-DNA junction [32]. In addition to the potential role in R-loop processing, RNase H1 has also been proposed to be involved in mitochondrial pre-rRNA processing by interacting with the mitochondrial protein P32, which slightly enhances the RNase H1 enzymatic activity [33].

Initiation of mitochondrial DNA replication has been suggested to resemble replication of the *E*. *coli* plasmid ColE1. In ColE1 replication, a transcript denoted RNAII associates with the template strand, forming an R-loop that serves as a primer for DNA synthesis [34, 35]. The ColE1 origin of replication is situated downstream of a guanine-rich stretch that is essential for both replication initiation and R-loop formation [35, 36]. For proper initiation of replication, RNAII has to be cleaved by *E*. *coli* RNase H, thereby creating a primer 3′-end that can be used by the replication machinery. The similarity between OriH of mtDNA and the ColE1 origin [27, 28], together with the recent in vivo findings of RNase H1 involvement in mtDNA synthesis, suggests that RNase H1 could be involved in primer processing in human mitochondria. To address this intriguing possibility, we here set out to reconstitute initiation of mtDNA replication in vitro.

## Results

### R-loop formation

**Fig 1:**
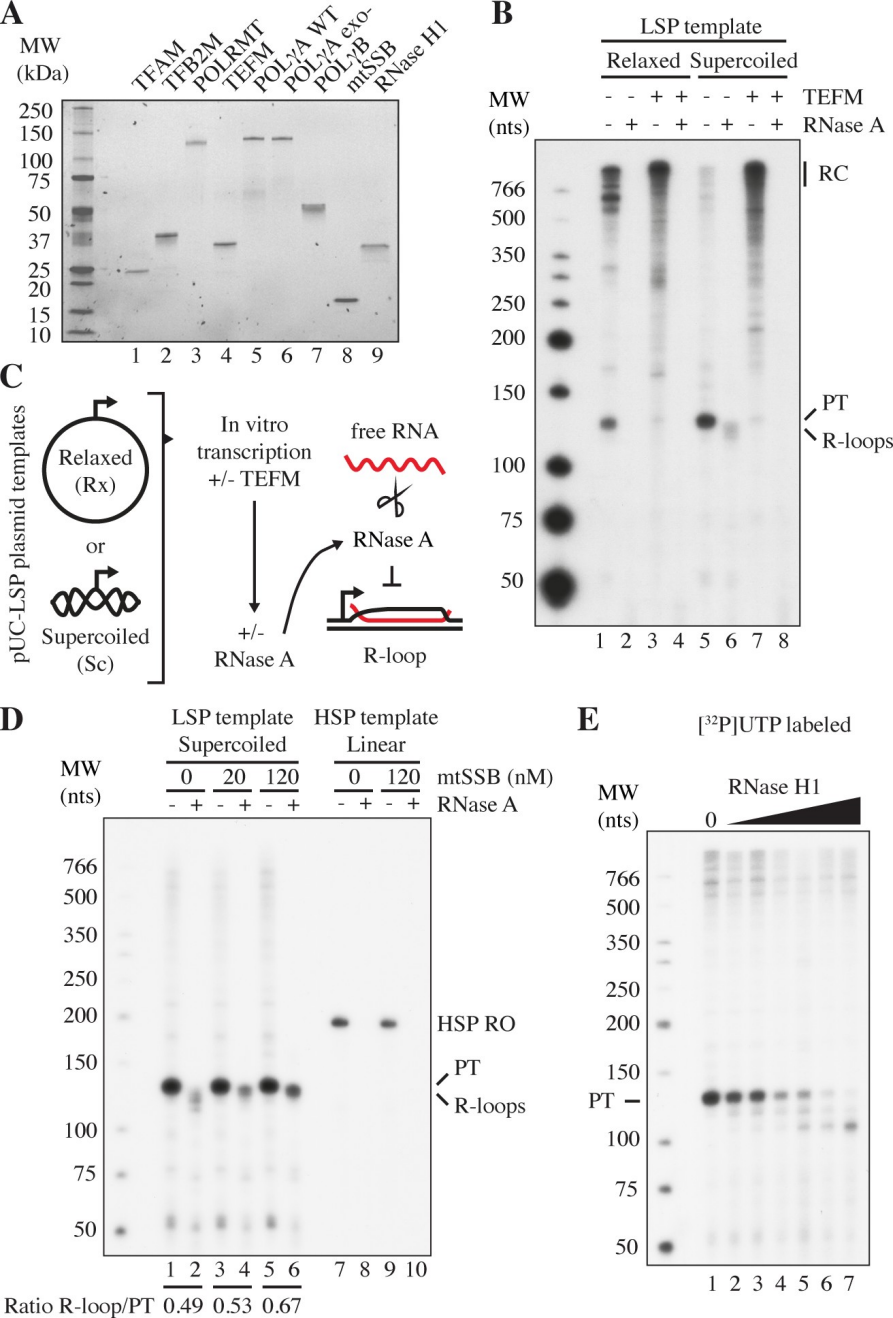
Factors affecting R-loop formation in vitro. A. Purified, recombinant proteins used in the present study visualized by Stain Free SDS-PAGE (Bio-Rad). B. In vitro transcription from LSP with POLRMT (20 nM), TFAM (200 nM) and TFB2M (60 nM). R-loops were formed and detected as described in panel C. TEFM (40 nM) was added to the indicated reactions. Products formed are labeled as followed: PT: transcripts prematurely terminated at CSBII; RC: longer transcripts formed by rolling circle transcription; and R-loops: transcripts unaffected by RNase A treatment (lane 6). The RNA was labeled by [^32^P]UTP incorporation. C. Reaction scheme for R-loop formation. A pUC18 plasmid containing an LSP insert, including the CSB region (pUC-LSP, S1 Table) was used. When indicated, the template was treated with topoisomerase I to relax supercoils. In vitro transcription was performed in the presence or absence of TEFM followed by the addition of 300 mM NaCl and RNase A to remove free RNA. D. Effects of mtSSB on in vitro transcription and R-loop formation. Templates used were supercoiled pUC-LSP (lanes 1-6) and as a control, linear pUC-HSP (lanes 7-10, see S1 Table for template sequence). mtSSB concentrations are indicated in nM. HSP RO: Run-off product of HSP transcription; PT: transcripts prematurely terminated at CSBII; and R-loops: transcripts unaffected by RNase A treatment. The ratio of R-loops/CSBII pre-terminated transcripts for each mtSSB concentration is indicated (see Materials and methods). E. R-loop formation was as in 1C, but without RNase A treatment. Increasing RNase H1 concentrations were added (0, 1, 2, 4, 8, 16 and 32 nM in lanes 1-7). PT indicates transcripts prematurely terminated at CSBII.

In vivo analysis has suggested that RNase H1 is required to process R-loops in vivo [30]. We decided to investigate this process in vitro and therefore set out to reconstitute R-loop formation. For our work, we used purified human mitochondrial transcription proteins (Fig 1A, lanes 1-4). As templates, we employed supercoiled or relaxed plasmids containing the LSP promoter and the downstream CSBI-III-region (pUC-LSP, S1 Table). On a relaxed template, a fraction of all transcription events initiated at LSP was prematurely terminated at CSBII (Fig 1B, lane 1) [26, 29]. This effect was stronger on a negatively supercoiled template, where nearly all transcription events were prematurely terminated at CSBII (Fig 1B, compare lanes 1 and 5). In agreement with previous reports, premature transcription termination was reduced by addition of the transcription elongation factor TEFM (Fig 1B, lane 7) [4, 5]. To detect if the pre-terminated transcripts remained as stable R-loops, we used an RNase A protection assay (Fig 1C). RNase A digests free RNA, whereas RNA associated with DNA is resistant to this nuclease at high salt concentrations. No R-loops were formed with relaxed LSP template (Fig 1B, lanes 2 and 4). In the reaction with the supercoiled template however, we observed RNase A-resistant products at sizes just below the transcript terminated at CSBII (Fig 1B, lane 6). This observation suggested to us that long R-loops were formed, possibly encompassing nearly the entire region from LSP to CSBII. Interestingly, R-loops were not detected when TEFM was added to the transcription reaction (Fig 1B, compare lanes 6 and 8).

In other systems, single-stranded DNA binding proteins can promote R-loop formation, probably by binding to the displaced DNA strand [37–39]. We therefore monitored the effects of mtSSB (Fig 1A, lane 8) on R-loop formation in vitro. Addition of mtSSB did not affect the overall transcription patterns on a negatively supercoiled LSP template (Fig 1D, compare lane 1 to lanes 3 and 5), but the R-loops formed in the presence of mtSSB appeared less processed and more uniform in size (Fig 1D, compare lane 2 to lanes 4 and 6). When 120 nM mtSSB was added nearly 70% of all pre-terminated transcripts end up as R-loops (Fig 1D, lanes 5-6), as compared to 50% in the absence of mtSSB (Fig 1D, lanes 1-2). To ensure that our results were not due to mtSSB binding directly to single-stranded RNA and protecting it from RNase A degradation, we also monitored the effects of mtSSB on transcription from a linearized HSP plasmid (pUC-HSP, S1 Table); a template that does not contain a downstream R-loop-forming region. The presence of mtSSB did not protect transcripts formed by HSP transcription from RNase A degradation (Fig 1D, lanes 7-10).

Finally, we investigated if RNase H1 (Fig 1A, lane 9) could process the R-loops. We found that RNase H1 gradually degraded R-loops in a concentration-dependent manner, generating shorter RNA species. (Fig 1E, compare lane 1 with lanes 2-7). The shorter RNA species may be involved in hybrid G-quadruplex formation, and therefore partially resistant to RNase H1 degradation [27]. The observed degradation of R-loops in vitro was in agreement with in vivo data indicating that RNase H1 can process mitochondrial R-loops [30].

### RNase H1 and mtSSB activate replication initiation from LSP R-loops

**Fig 2:**
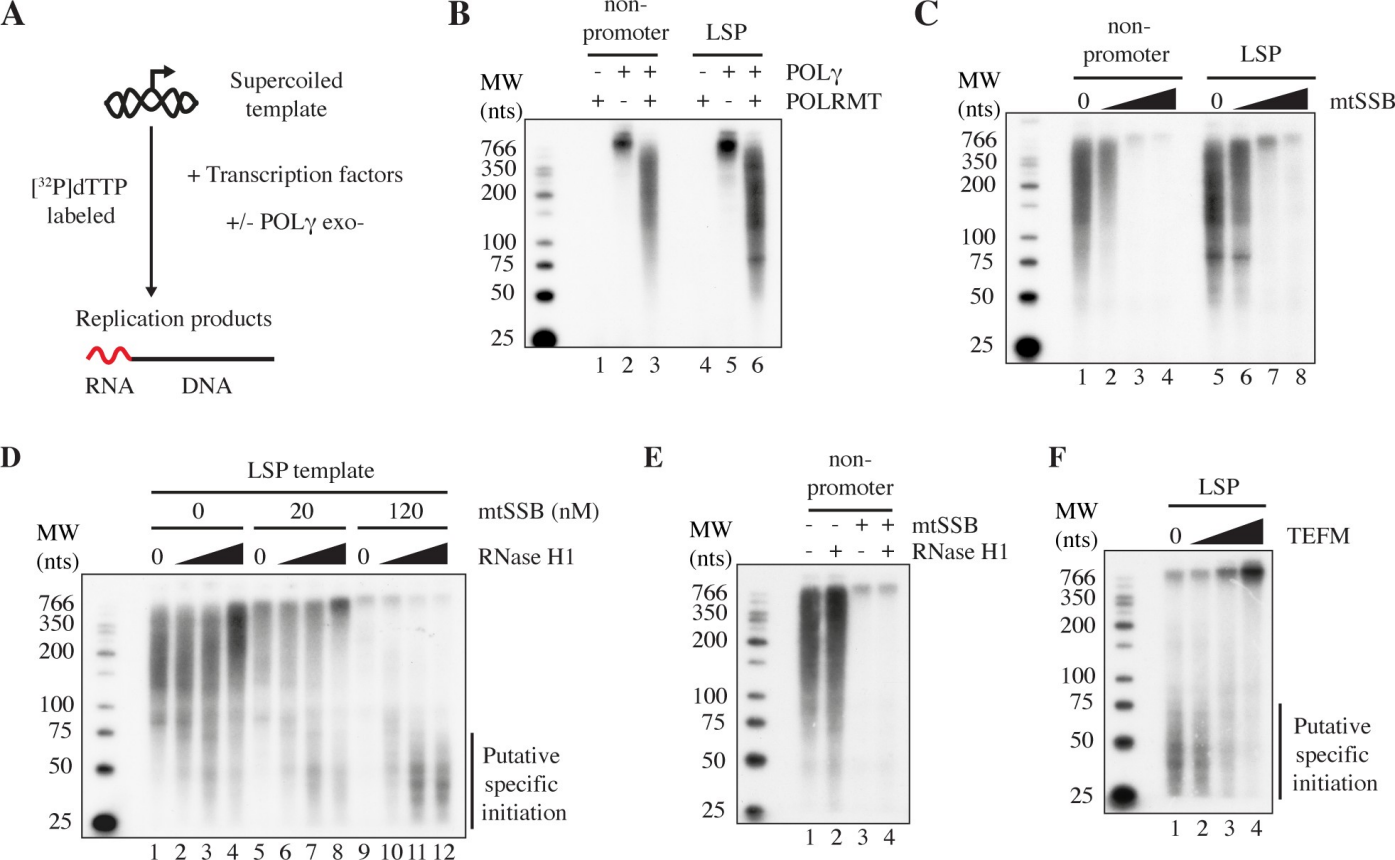
Replication initiation on supercoiled templates. A. Reaction scheme for replication initiation reactions combining mitochondrial transcription and replication proteins with supercoiled templates. B. Reactions as described in panel A, with supercoiled pUC19 (lanes 1-3) or pUC-LSP (lanes 4-6) in the presence or absence of POLγ and POLRMT. DNA synthesis products were labeled by [^32^P]dTTP. Products at the top of the gel are likely due incorporation of radioactive nucleotides at plasmid nicks (“nick translation”), since these products are observed also with POLγ only (Fig 2B, lanes 2 and 5). C. Reactions described in panel A with supercoiled pUC19 (lanes 1-4) or pUC-LSP (lanes 5-8) in the presence of both POLγ and POLRMT. Increasing amounts of mtSSB were added (0, 8, 32, 128 nM in lanes 1-4 and 5-8 respectively). DNA synthesis products were labeled by [^32^P]dTTP. D. POLγ-dependent DNA synthesis from processed R-loops in reactions as in Fig 2A using the pUC-LSP template. DNA synthesis products were labeled by [^32^P]dTTP. RNase H1 (0.5, 2 and 8 nM in lanes 1-4, 5-8 and 9-12 respectively) and mtSSB (20 nM in lanes 5-8 or 120 nM in lanes 9-12) were added when indicated. DNA synthesis products formed in presence of RNase H1 and mtSSB are indicated (Putative specific initiations). E. POLγ-dependent DNA synthesis with a pUC19 template. DNA synthesis products were labeled by [^32^P]dTTP. RNase H1 (2 nM) and mtSSB (120 nM) were added as indicated. F. TEFM abolishes POLγ-dependent DNA synthesis from processed R-loops. Reactions were performed as in panel A and B, with pUC-LSP template in the presence of 120 nM mtSSB, 2 nM RNase H1 and increasing concentrations of TEFM (0, 4, 20 and 100 nM in lanes 1-4 respectively). RNase H1 dependent DNA synthesis products are indicated (Putative specific initiations).

It has been suggested that transcripts prematurely terminated at CSBII may be used to prime initiation of mtDNA replication [26]. We therefore decided to analyze if POLγ (Fig 1A lanes 5-7) can use the R-loops formed in vitro as primers for initiation of DNA synthesis. We first investigated if POLγ can use random RNA primers to initiate DNA synthesis on dsDNA. To this end, we utilized POLRMT’s ability to initiate transcription and produce short RNA molecules on negatively supercoiled dsDNA even in the absence of a promoter sequence [40]. In our experiments, we used an exonuclease deficient version of POLγ (exo-), since this protein has strand displacement activity and can use dsDNA as a template even in the absence of a DNA helicase [41]. To monitor DNA synthesis we performed the reactions in the presence of [^32^P]dTTP (Fig 2A). When both POLRMT and POLγ were added simultaneously to a negatively supercoiled dsDNA template (without LSP), we observed formation of [^32^P]dTTP-labeled DNA products ranging in size from 50 to 700 nts (Fig 2B, lane 3). We also did the experiment with a template containing LSP but did not find any major effect of its presence (Fig 2B, lane 6). When we repeated the experiment in the presence of increasing amounts of purified mtSSB, replication products disappeared (Fig 2C, lanes 2-4 and 6-8). POLγ can thus initiate DNA synthesis from random primers generated by POLRMT and mtSSB represses this activity. The repressive effect of mtSSB is probably due to its ability to prevent transcription initiation by POLRMT on ssDNA [15, 42].

We next monitored the effects of RNase H1 on replication. Using the LSP template, in the absence of mtSSB, we again observed abundant replication products ranging in size from 50 to 700 nts and we observed a mild stimulatory effect at high levels of RNase H1 (Fig 2D, compare lanes 1 and 4). Again, these non-specific products were reduced in the presence of low levels of mtSSB (20 nM, Fig 2D, lane 5) and abolished at high concentrations of mtSSB (120 nM, Fig 2D, lane 9). Interestingly, addition of increasing levels of RNase H1 in the presence of mtSSB caused the formation of new group of DNA products, ranging in size between 25 and 100 nts (Fig 2D, lanes 11-12). We hypothesized that these products could be DNA replication events initiated from processed R-loops formed by transcription from LSP. To address this possibility, we repeated the experiments with a template lacking LSP. On this template, RNase H1 had no apparent effects on DNA synthesis and no short DNA products were observed when RNase H1 was added together with mtSSB (Fig 2E, lanes 1-4). To further demonstrate that priming was dependent on R-loops, we performed our experiments with the LSP-containing template in the presence of TEFM, which at high concentrations prevents R-loop formation. Increasing amounts of TEFM reduced the products in the size range between 25 and 100 nts, thus suggesting that these shorter replication products were indeed dependent on R-loop formation downstream of LSP (Fig 2F, lanes 1-4). In the presence of higher concentrations of TEFM, a high molecular weight species started to accumulate (Fig 2F, lane 4), which may be due to some longer RNA molecules being used as non-specific primers for replication. Interestingly, even if TEFM reduces replication initiation, it does not completely abolish the reaction. Shorter replication products were observed even when TEFM was present in excess (Fig 2F, lane 4).

### Initiation of replication from processed LSP R-loops

**Fig 3:**
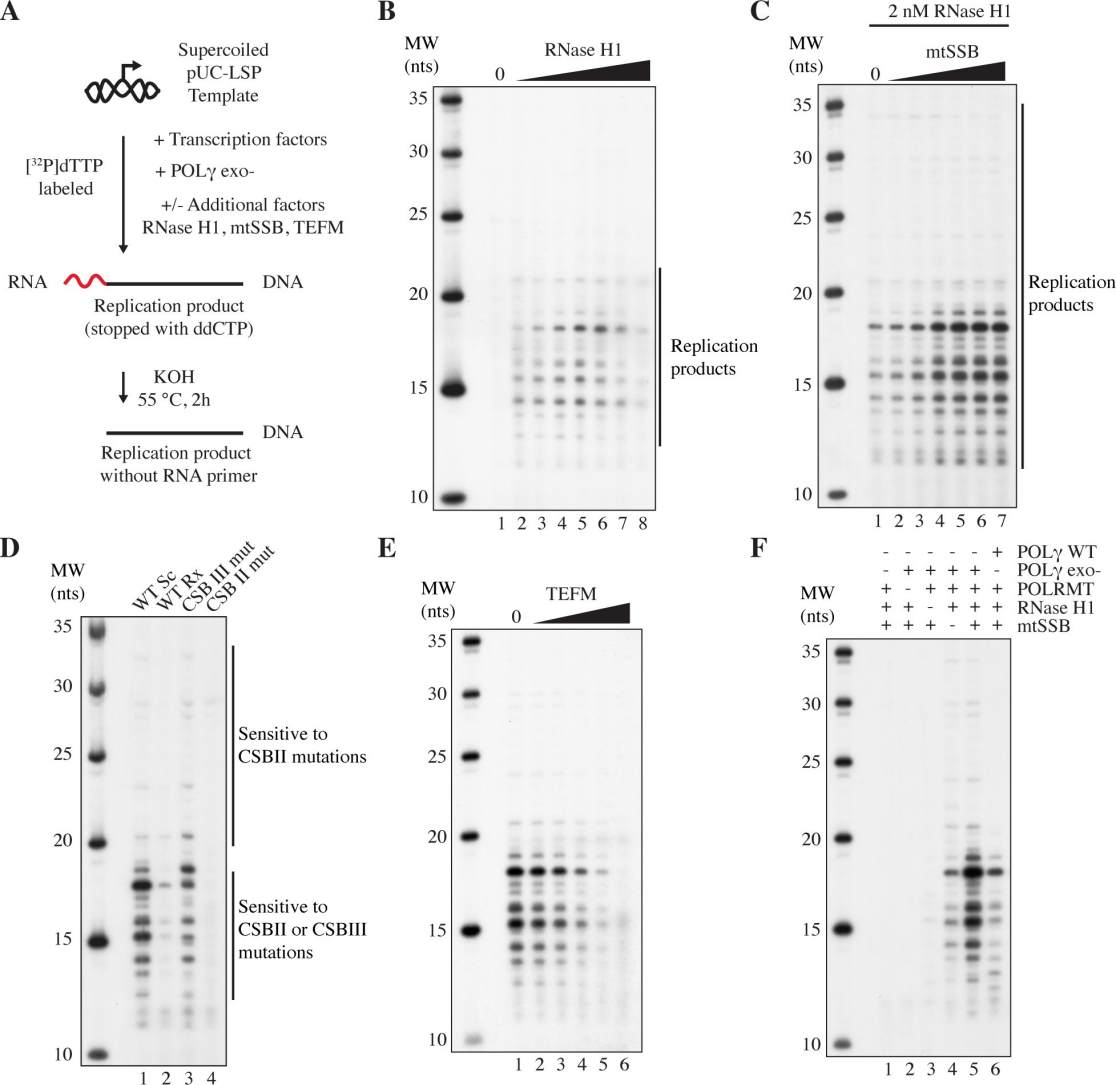
RNase H1 and mtSSB activate R-loop dependent replication initiation. A. Reaction scheme for R-loop-specific initiation of replication. Replication products were labeled by [^32^P]dTTP and DNA synthesis was terminated by ddCTP incorporation. KOH treatment was used to remove any ribonucleotide primer residues. Products were separated on denaturing sequencing gels. The same basic reaction was performed in panels B-F, with the indicated modifications. B. Replication initiation in the absence of mtSSB. RNase H1 was added as indicated (lane 2-8, 0.25, 0.5, 1, 2, 4, 8 and 16 nM respectively). C. Replication initiation with constant amounts of RNase H1 (2 nM). mtSSB was added as indicated (lane 2-7, 2.5, 5, 10, 20, 40 and 80 nM respectively). D. Replication initiation with pUC-LSP and mutant derivatives thereof; WT, supercoiled (lane 1), WT relaxed (lane 2), CSBIII mutant (lane 3, see S1 Table), and CSBII mutant (lane 4, see S1 Table). Initiation events sensitive to CSBIII and CSBII, or only to CSBII, are indicated. E. Replication initiation in the absence (lane 1) or presence of increasing amounts of TEFM (5, 10, 20, 40 and 80 nM in lanes 2-6 respectively). F. Replication initiation with WT POLγ and/or the indicated factors. All reactions also contain TFAM, TFB2M and POLγB.

To further verify that the 25-100 nts replication products we had observed indeed originated from an LSP R-loop primer, we utilized the fact that the LSP R-loop region only contains a few guanines in the template strand. By adding ddCTP to our reactions we could therefore generate short specific length replication products. Before gel analysis, the replication products were treated with potassium hydroxide (KOH) to remove any RNA residues [43, 44] (Fig 3A). As expected, we could not observe any DNA synthesis in the absence of RNase H1 (Fig 3B, lane 1), again demonstrating that the unprocessed R-loops do not function as primers. Upon addition of RNase H1, we observed replication products with sizes between 12 and 21 nts (Fig 3B, lanes 2-8). The highest levels of DNA synthesis were observed at 2 nM of RNase H1 (Fig 3B, lane 5). Furthermore, we found that mtSSB had a strong stimulatory effect on the reactions (Fig 3C, compare lane 1 to lanes 2-7). By introducing mutations that changed the length of C-less stretches downstream of LSP, we could verify that replication products observed after incubation with ddCTP was due to initiation near CSBII and CSBIII (S1A–S1C Fig). Combined, our experiments therefore support the hypothesis that RNase H1 is required to process R-loops for primer formation and that mtSSB stimulates the process. For all subsequent experiments, we used an mtSSB concentration of 40 nM together with 2 nM of RNase H1 (Fig 3C, lane 6).

### Replication initiation depends on the CSB-anchored R-loops

We next examined the effects of differing template topology and CSB region mutations on replication initiation. Relaxation of the DNA template, which impairs R-loop formation, almost completely abolished replication initiation (Fig 3D, compare lanes 1 and 2). When CSBIII was mutated, the levels of most replication products shorter than 20 nts were slightly decreased, whereas longer products were left unaffected (Fig 3D, compare lanes 1 and 3). When CSBII was mutated all replication initiation events disappeared (Fig 3D, compare lanes 1 and 4). These results confirm that the LSP R-loops are essential for replication initiation and demonstrate that whereas the CSBII element is crucial for successful primer formation, CSBIII only has a minor effect on initiation (Fig 3D, compare lanes 3 and 4).

Next, we examined the effects of TEFM on initiation of DNA synthesis (Fig 3E). At lower concentrations, TEFM did not affect replication initiation. Interestingly, robust levels of initiation were observed even at equimolar concentrations of POLRMT and TEFM (Fig 3E, lane 4). Not even at very high TEFM concentrations (molar ratio 4:1 relative POLRMT) could we observe a complete inhibition of replication initiation (Fig 3E, lane 6).

Our experiments so far had been performed with exonuclease deficient POLγ. We now repeated the experiments with WT POLγ (Fig 1A, lane 5). As previously demonstrated, initiation of DNA synthesis required the presence of POLγ, POLRMT, and RNase H1 (Fig 3F). Furthermore, the reaction was stimulated by the addition of mtSSB (Fig 3F, compare lanes 4 and 5). The POLγ WT enzyme produced the same products as seen with POLγ exo- (Fig 3F, compare lanes 5 and 6), although the levels of replication products were lower, probably due to the weaker strand displacement activity of the WT enzyme.

### Impaired RNase H1 activity changes replication initiation in vivo

**Fig 4:**
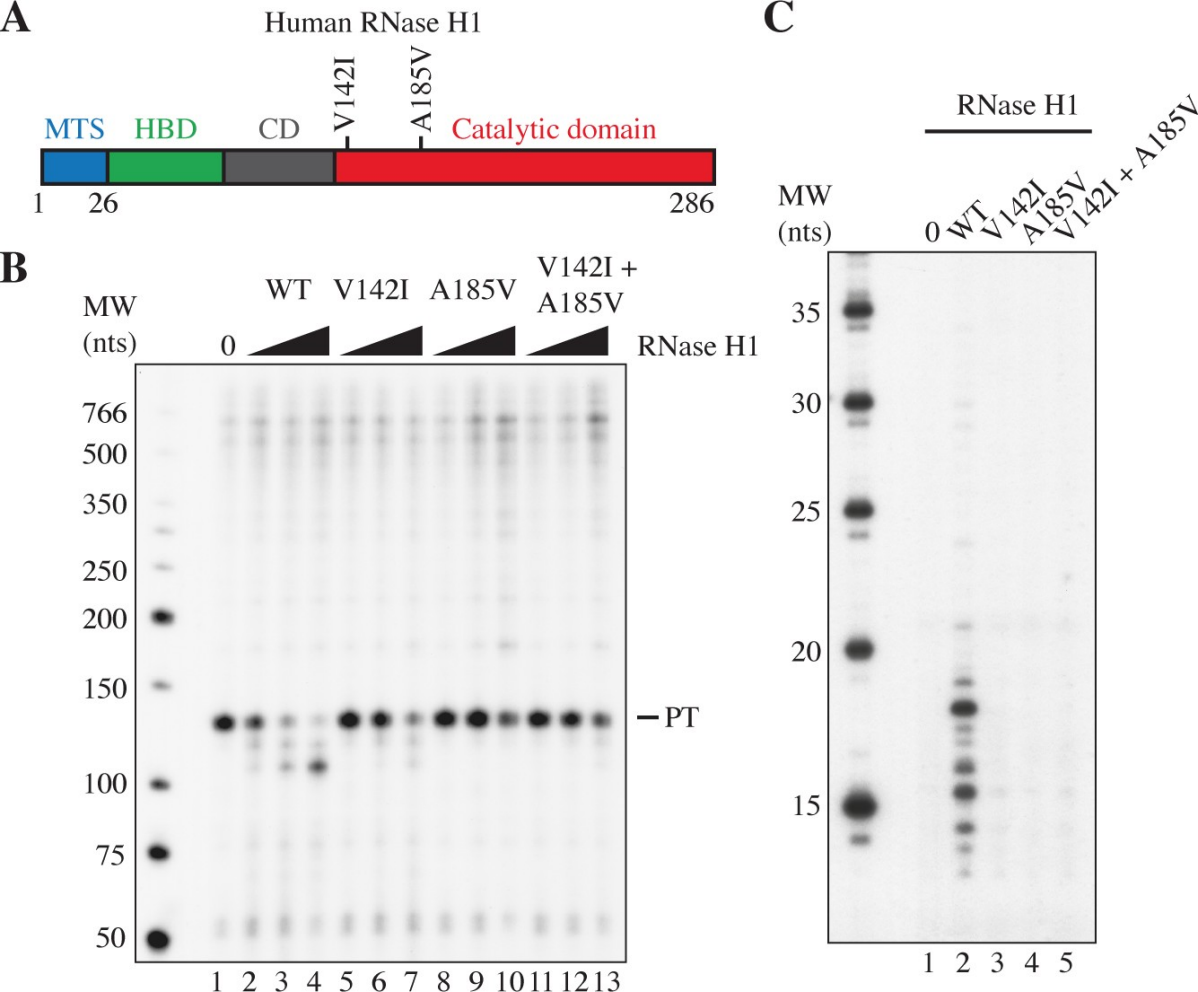
RNase H1 disease mutants are unable to support primer processing and DNA replication initiation. A. Schematic representation of human RNase H1 with the V142I and A185V mutations indicated. The mitochondrial targeting signal (MTS) is shown in blue, the hybrid binding domain (HBD) in green, the connection domain (CD) in grey and the catalytic domain in red. B. R-loop processing by RNase H1 mutant enzymes. The reactions were performed as in Fig 1E. WT, V142I or A185V RNase H1 enzymes were added at 2, 8 and 32 nM in lane 2-10. Reactions in lanes 11-13 contained both V142I and A185V mutants with total enzyme concentration still at 2, 8 and 32 nM. Lane 1 is a no RNase H1 control. PT indicates transcripts prematurely terminated at CSBII. C. Replication initiation reaction as described in Fig 3, with 40 nM mtSSB and 2 nM RNase H1 WT or mutants as described in panel B.

Based on our in vitro observations, we wanted to specifically analyze the effects of reduced RNase H1 activity on mtDNA replication initiation in mammalian cells. To this end, we decided to study the effects of disease causing mutations in *RNASEH1*, which are associated with adult-onset mitochondrial encephalomyopathy [45]. First, we expressed two disease-causing mutant forms of RNase H1, RNase H1:V142I and RNase H1:A185V (Fig 4A) as recombinant proteins and analyzed their effects on R-loop processing. Both RNase H1:V142I and RNase H1:A185V, alone or in combination (Fig 4B, lanes 5-13), displayed impaired R-loop processing activity compared to WT RNase H1 (Fig 4B, lanes 2-4). As a consequence, the mutant RNase H1 proteins could not support origin-specific initiation of DNA replication in vitro (Fig 4C and S2 Fig).

**Fig 5:**
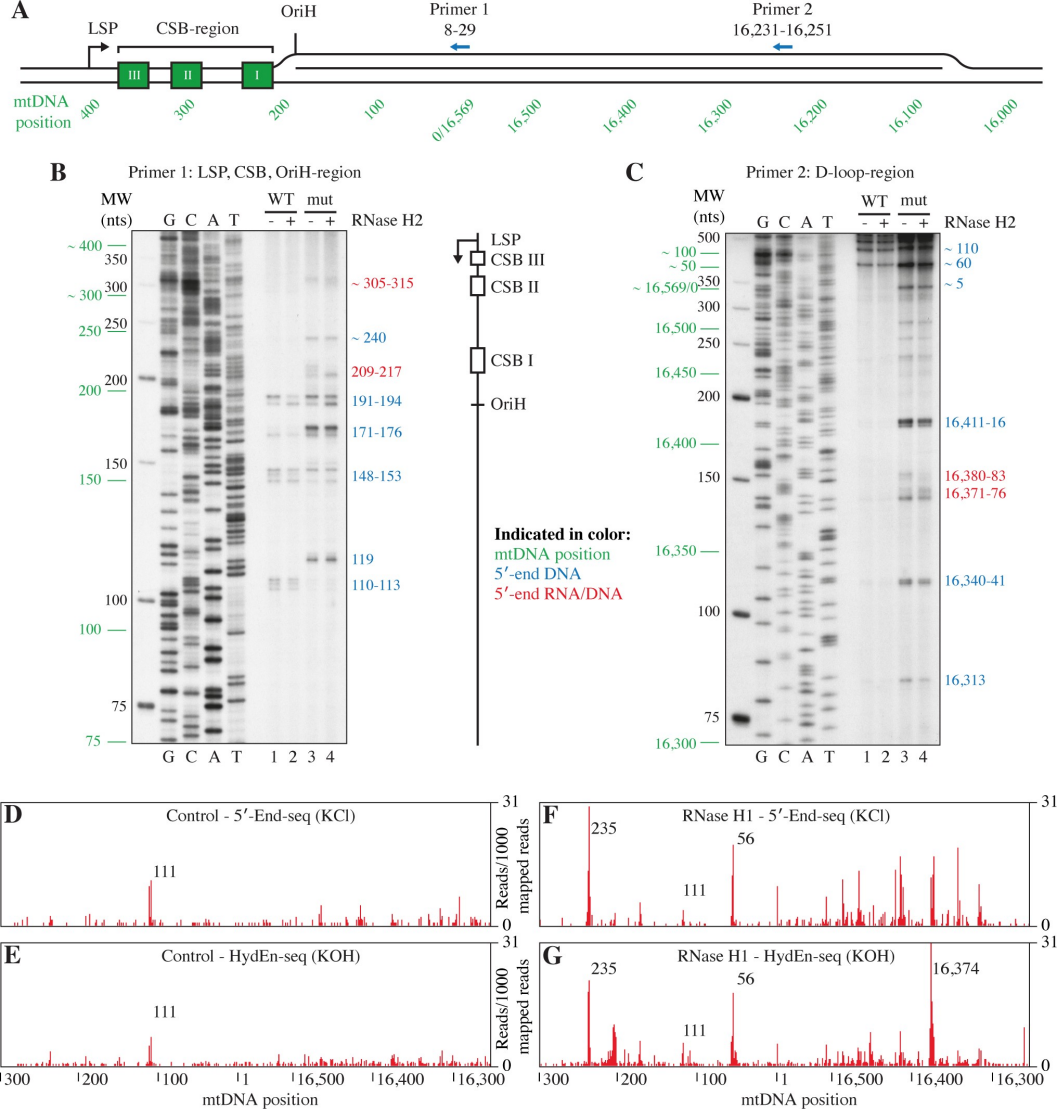
DNA replication initiation defects in RNase H1 deficient cells. A. Schematic representation of the control region of mtDNA with the positions of primer extension primers 1 and 2 indicated. B. Primer extension of mtDNA from control and RNase H1 patient cells with primer 1 (corresponding to mtDNA positions 8-29, see panel A). Primer 1 was annealed to mtDNA from control (WT) and RNase H1 patient (mut RH1) cells and extended over the OriH, CSB and LSP regions with Taq DNA polymerase (see method details). Control cell DNA (WT) in lanes 1-2 and RNase H1 patient cell DNA in lanes 3-4. Untreated DNA in lanes 1 and 3, and RNase H2 treated DNA in lanes 2 and 4. The NEB LMW ladder is indicated in black, mtDNA positions in green, mapped 5′-ends in blue and mapped RNA to DNA transition points in red. G, C, A and T sequencing ladders are found on the left-hand side. A schematic representation of the control region with OriH, CSBI-III and LSP is shown on the right-hand side. C. Primer extension of mtDNA from control and RNase H1 patient cells with primer 2 (corresponding to mtDNA positions 16,231-16,251, see panel A). Sample loading and colored indications as in panel B. D. 5′-end sequencing (5′-End-seq) of control cell mtDNA. E. Hydrolytic end sequencing (HydEn-seq) of control cell mtDNA to map 5′-ends with attached ribonucleotides. F. 5′-end sequencing (5′-End-seq) of RNase H1 patient cell mtDNA. G. Hydrolytic end sequencing (HydEn-seq) of RNase H1 patient cell mtDNA to map 5′-ends with attached ribonucleotides.

Second, we used fibroblasts isolated from a patient with two heterozygous *RNASEH1* mutations, the V142I mutation (see Fig 4A), and a second mutation, (R157*), causing a truncated form of the enzyme lacking the active site. In our experiments, we used primer extension with DNA isolated from these RNase H1-deficient patient cells and a wild-type (WT) control to map initiation sites for mtDNA synthesis in vivo. We designed one primer to detect 5′-ends close to OriH and the CSB-region (primer complementary to H-strand positions 8-29, Fig 5A, Primer 1) and another to detect 5′-ends further downstream, in the D-loop region (primer complementary to H-strand positions 16,231-16,251, Fig 5A, Primer 2). The isolated DNA was analyzed before and after treatment with E. coli RNase H2, which will specifically degrade the RNA part of the covalently linked RNA-DNA molecule and thereby allow for exact mapping of RNA to DNA transitions. In control cells, 5′-ends were detected at mtDNA positions 191-194, 171-176, 148-153 and 110-113 (Fig 5B, lane 1) as well as around mtDNA position 60 (Fig 5C, lane 1). The observed 5′-ends were not altered by RNase H2 treatment, demonstrating that they were fully processed i.e. had no RNA residues attached (Fig 5B and 5C, compare lanes 1 and 2). Using mtDNA isolated from RNase H1 deficient cells, we observed a strong increase in 5′-ends. Some 5′-ends were similar to, but sometimes more abundant, than the products observed in WT cells at positions 191-194, 148-153, 171-176 and around position 60 (Fig 5B and 5C, lane 3). We also noted a range of new 5′-ends not present in WT cells at positions ~305-315, ~240, 209-217 and 119 (Fig 5B, lane 3) and throughout the D-loop region (Fig 5C, lane 3). Interestingly, at least three of these new 5′-ends were altered upon RNase H2 treatment suggesting that they represented new RNA to DNA transition sites not present in the WT control. These new sites were located in the CSB region at positions 305-315 and 209-217 (Fig 5B, compare lanes 3 and 4), and in the D-loop, at positions 16,371-16,383 (Fig 5C, compare lanes 3 and 4). Our data thus suggested that loss of RNase H1 activity caused initiation of mtDNA synthesis from multiple sites not used in WT cells.

To further support our findings, we also used an alternative method to identify 5′-ends and RNA to DNA transitions. We performed 5′-End-seq and HydEn-seq on the mtDNA from control and patient cells deficient in RNase H1. The 5′-End-seq method will detect all 5′-ends of DNA, including DNA molecules with ribonucleotides on the 5′-end. In contrast, HydEn-Seq will only detect DNA 5′-ends, since any RNA residues will be chemically removed [43]. By comparing results from 5′-End-seq and HydEn-seq, it is thus possible to identify RNA to DNA transition sites. The major DNA 5′-end in the control cells was mapped to position 111 (Fig 5D). There were no major differences between the 5′-End-seq and HydEn-seq samples indicating that no RNA-DNA transitions are found in this region in control cells (Fig 5D and 5E). We observed more reads in the control region of RNase H1-deficient patient cells (Fig 5F) compared to control cells, in agreement with the increased 5′-ends found by primer extension. Interestingly, 5′-ends of DNA were clearly shifted when compared to the control cells. New large peaks appeared around positions 235 and 56 and a region spanning 16,569/0 to 16,300 (Fig 5F). When comparing the 5′-End-seq to the HydEn-seq data for the peaks in the 16,569/0 to 16,300 region, it was clear that all the peaks decreased in the HydEn-seq data, with the exception of a peak at 16,374, which was slightly increased (Fig 5G). One region with new peaks also appeared in the HydEn-seq data, around position 200. In conclusion, the sequencing results agreed with the findings obtained by primer extension. The data from these two methods revealed an overall increase in 5′-ends in the control region of RNase H1-deficient patient cells as well as new RNA to DNA transition points. In conclusion, loss of RNase H1 activity leads to initiation of mtDNA synthesis at multiple sites not used in WT cells.

## Discussion

It has long been recognized that mammalian mtDNA replication is initiated at OriH [19–21]. Studies in mitochondrial extracts have also demonstrated that R-loops are formed in the region downstream of LSP and linked these structures to priming of mtDNA replication in the OriH-region [25]. The precise mechanisms of the proposed model have remained obscure, in part because of technical limitations. For instance, mitochondria cannot be transfected, a shortcoming that has prevented a detailed structural characterization of OriH. We here use an alternative approach to study OriH function, employing purified, recombinant proteins and defined DNA templates. Our work builds on recent in vivo observations, which demonstrated that RNase H1 is required for R-loop processing and mtDNA replication in a mouse knockout model system [30].

Early reports suggested that the LSP transcript involved in R-loop formation must be cleaved to form the 3′-OH termini required for initiation of replication at OriH. RNase MRP was identified as a candidate for this process [46–49], but the idea was later abandoned, since experimental evidence argued against the existence of RNase MRP in mitochondria [50–52]. As demonstrated here, the R-loops formed by LSP-transcription are instead processed by RNase H1 and the 3′-ends formed are used to prime mtDNA synthesis by POLγ. The process is stimulated by mtSSB, which acts to stabilize R-loops, most likely by binding to the displaced DNA strand. In addition, mtSSB prevents non-promoter-specific initiation of transcription from single-stranded stretches of DNA [15], thereby restricting replication initiation to the OriH-region. Our findings receive support from previous work, which has demonstrated that *Rnaseh1* deletion in mice leads to mtDNA depletion [53] and that disease-causing mutations in *RNASEH1* impair mtDNA replication [45, 53, 54].

In our work, we found that DNA replication in vitro was mainly initiated from the CSBII and CSBIII regions. The observed RNA to DNA transitions mapped in our recombinant system therefore correlate with the RNA to DNA transitions previously mapped in mtDNA by David Clayton and colleagues [19, 20, 47, 48]. In later papers, primer extension and ligation mediated PCR was used to map RNA to DNA transition points and only one of the mapped regions, that downstream of CSBII, was identified as a replication initiation site in vivo [26, 29]. What was not known at the time of these later studies was that the CSBII sequence forms strong G-quadruplex structures in both DNA and RNA [27, 28]. Many DNA polymerases have problems bypassing such G-quadruples and primer extension can in fact be used to detect these non-B-form DNA structures [55]. The way that these experiments were devised may therefore have correctly mapped replication initiation sites located downstream of CSBII, but failed to reach 5′-ends on nascent mtDNA located upstream of the G-quadruplex-forming CSBII region, thereby missing initiation events near CSBIII.

Interestingly, mutations that reduce RNase H1 activity in vivo lead to an increase of 7S DNA levels [45]. We believe that this effect is due to unregulated initiation of 7S DNA synthesis. In support of this notion, our primer extension and 5′-end sequencing results show an overall increase in free 5′-ends and identified multiple new RNA to DNA transition sites. Apparently, RNase H1 is required to restrict initiation of 7S DNA and mtDNA synthesis to OriH. The enzyme removes all RNA hybridized to the DNA template, with the exception of the RNA-loop molecules in the CSB region, which after RNase H1 processing can be used as primers. In the absence of RNase H1, 7S DNA synthesis is instead initiated from multiple locations, possibly from any RNA molecule with a 3′-end hybridized to the DNA template, leading to unregulated initiation of 7S DNA synthesis and an increase in overall 7S DNA levels. In contrast, the mtDNA levels remain largely unchanged in *RNASEH1* mutant cells. This observation is in agreement with the notion that regulation of mtDNA levels is not correlated to 7S DNA levels, but takes place at the end of the D-loop [56]. At this place, there is a regulated switch between abortive (7S DNA) and genome length mtDNA replication, which explains why 7S DNA can be strongly up regulated, whereas mtDNA levels remain unaffected. The overall problem in the case of RNase H1 deficiency could instead be related to downstream events, such as DNA replication termination and mtDNA segregation, as the newly synthesized mtDNA will have shifted 5′-ends and thus also replication end points.

TEFM prevents premature transcription termination at CSBII, which led to the suggestion that this protein can function as a regulator of primer formation, controlling the switch between transcription and mtDNA replication [4, 5]. In support of this claim, addition of TEFM reduces R-loop formation in vitro. The effects of TEFM on replication initiation are less dramatic, as robust initiation is seen even when POLRMT and TEFM are present at equimolar levels. This observation does not necessarily argue against the idea that TEFM acts to regulate the switch between transcription and replication. It is still possible that variations in TEFM concentrations will affect the relative levels of primer formation and transcription elongation in vivo. However, TEFM does not cause an all or nothing effect, since the protein may be present at relatively high concentrations without abolishing replication initiation. Also arguing against an essential role for TEFM in regulating primer synthesis, depletion of this factor in cell lines does not lead to changed levels of mtDNA [3]. Clearly, more work is required to elucidate the precise role of TEFM in the regulation of mtDNA replication initiation.

RNase H1 processing of R-loops may be influenced by a multitude of factors. The G-quadruplex structure formed between RNA and the non-template DNA strand at CSBII can reduce RNase H1 cleavage [27]. RNase H1 activity can also be influenced by other proteins. There could be e.g. direct protein interactions between RNase H1 and the transcription or replication proteins. In addition, RNase H1 has been shown to interact with the mitochondrial protein P32 and that this interaction significantly enhances the cleavage activity of RNase H1 on heteroduplex templates. If P32 also affects primer formation is not known [33]. We will address these intriguing possibilities in future studies.

**Fig 6:**
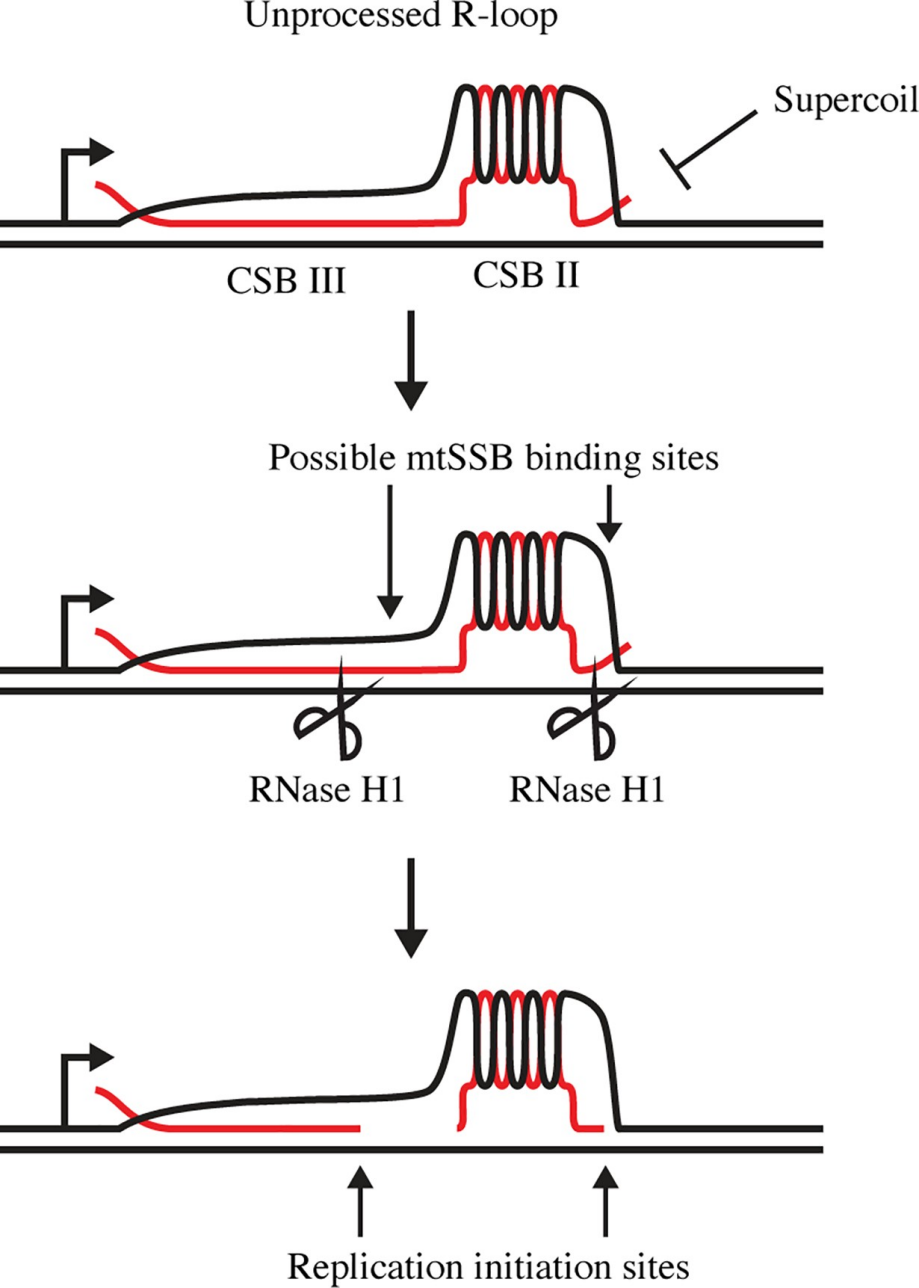
A simplified model for replication primer formation and replication initiation downstream of LSP. Transcription termination at CSBII leads to the formation of an R-loop, which is stabilized by a G-quadruplex structure formed between the nascent RNA and the G-rich non-template DNA strand. To be used as a primer for mtDNA synthesis, the R-loop must first be processed by RNase H1 to generate 3′-ends, from which POLγ can initiate DNA synthesis. Both R-loop formation and DNA replication initiation are stimulated by mtSSB.

In conclusion, we here present a mechanism for primer formation and initiation of DNA replication in human mitochondria (as shown in Fig 6). As previously suggested [20, 23, 25], there are indeed striking similarities between replication initiation in mammalian mitochondria and the process described for the ColE1 plasmid. Both systems depend on promoter driven transcription, R-loop formation and primer processing. Important aspects of our model are supported by in vivo evidence, but more work is clearly needed to validate our ideas and refine the proposed mechanisms. Of special importance will be to clarify how the switch between transcription and primer synthesis is regulated.

## Materials and methods

### Protein purification

RNase H1 was expressed using the baculovirus system (Sf9 cells). The protein coding sequence was PCR-amplified from human cDNA and cloned into the pBacPAK9 vector (Clontech). The construct lacked the N-terminal MTS (amino acids 1-26), and carried a C-terminal 6×His-tag. Recombination and cell infection was performed as described in the BacPAK manual (Clontech). The protein was expressed and purified as previously described for transcription proteins [5]. All other recombinant proteins were expressed and purified as described previously [5, 41].

### Template preparation

All templates used were either empty pUC19 plasmids or mitochondrial DNA sequences with or without modifications cloned in pUC18. A list of all templates used in this study can be found in S1 Table. All supercoiled templates were carefully prepared with QIAprep Spin Miniprep Kit (QIAGEN) and kept at 4°C. To produce relaxed templates, supercoiled plasmids were treated with Topoisomerase I (New England Biolabs).

### In vitro transcription

All transcription reaction volumes were 25 μL and contained 25 mM Tris-HCl pH 8.0, 10 mM MgCl_2_, 64 mM NaCl, 100 μg/mL BSA, 10 mM DTT, 400 μM ATP, 150 μM GTP, 150 μM CTP 10 μM UTP, 0.027 μM α-[^32^P]UTP (3000 Ci/mmol), 4 nM of indicated plasmid template, 20 nM POLRMT, 200 nM TFAM, 60 nM TFB2M, and 40 nM TEFM where indicated. The reactions were incubated at 32°C for 5 min and stopped by the addition of 200 μL stop buffer (10 mM Tris-HCl pH 8.0, 0.2 M NaCl, 1 mM EDTA, 100 μg/mL glycogen (Roche) and 100 μg/mL proteinase K (Ambion)) followed by incubation at 42°C for 45 min. The transcripts were recovered by ethanol precipitation and the pellets were dissolved in 20 μL gel loading buffer (98% formamide, 10 mM EDTA, 0.025% xylene cyanol FF, and 0.025% bromophenol blue) and heated at 95°C for 3 min. The samples were analyzed on 4% denaturing polyacrylamide gels (1 × TBE and 7 M urea) followed by exposure on photo film. Low Molecular Weight DNA Ladder (NEB) was used as a size marker. All experiments were performed multiple (>3) times with similar results and each figure shows a representative gel image for that experiment.

### R-loop detection by RNase A digestion

For R-loop detection, 1.5 μL of 5.0 M NaCl was added to each sample after the in vitro transcription reaction, followed by addition of 250 ng of RNase A (ThermoFisher Scientific) and incubation at 32°C for 5 min. The reactions were stopped and evaluated as the regular transcription reactions. Quantifications of R-loops were performed using ImageJ software (https://imagej.nih.gov/ij/). The intensity of R-loops larger than 100 nts was divided by the intensity of the CSBII transcript of the same reaction, to obtain a ratio indicating how efficient R-loop formation was. All experiments were performed multiple times with similar results and each figure shows a representative gel image for that experiment.

### Replication initiation

All reaction volumes were 25 μL and contained 25 mM Tris-HCl pH 8.0, 10 mM MgCl_2_, 50 mM NaCl, 100 μg/mL BSA, 10 mM DTT, 400 μM ATP, 150 μM GTP, 150 μM CTP 150 μM UTP, 100 μM dATP, 100 μM dGTP, 100 μM dCTP or ddCTP as indicated, 10 μM dTTP, 0.027 μM α-[^32^P]dTTP (3000 Ci/mmol), and 8 nM of indicated template. In the case of RNA labeling the concentration of dTTP were 100 μM and UTP concentrations were 10 μM UTP and 0.027 μM α-[^32^P]UTP (3000 Ci/mmol). All reactions (unless otherwise stated) contained 200 nM of TFAM, 60 nM of TFB2M, 20 nM of POLRMT, 20 nM of D274A POLγA exo- (or WT in Fig 3F lane 6) and 40 nM POLγB. RNase H1 was added at 2 nM and mtSSB was added at 40 nM unless otherwise stated. The reactions were incubated at 32°C for 30 min. Experiments with dCTP were stopped and evaluated as transcription reactions. Experiments with ddCTP were stopped by the addition of 5 μL stop buffer (to a final concentration of 10 mM Tris-HCl pH 8.0, 0.2 M NaCl, 1 mM EDTA, 660 μg/mL glycogen (Roche) and 100 μg/mL proteinase K (Ambion)) followed by incubation at 42°C for 45 min. The reactions were treated with KOH (300 mM) for 2 hrs at 55°C. The samples were recovered by ethanol precipitation in the presence of 0.5 volumes ammonium acetate (7.5 M), dissolved in 10 μL gel loading buffer (98% formamide, 10 mM EDTA, 0.025% xylene cyanol FF, and 0.025% bromophenol blue), heated at 95°C for 3 min. The products were analyzed on 6% denaturing polyacrylamide gels (1 × TBE and 7 M urea) for samples with dCTP or 12% denaturing polyacrylamide sequencing gels (1 × TBE and 7 M urea) for samples with ddCTP. The gels were exposed on photo film. All experiments were performed multiple times with similar results and each figure shows a representative gel image for that experiment.

### Cell culture and DNA purification

Patient and control fibroblast cells were grown in DMEM GlutaMAX medium, supplemented with 10% FBS, PEST and 10 μg/ml uridine in 5% humidified atmosphere at 37°C. Approximately 5 × 10^6^ cells were collected and lysed for 30 min at 42°C in lysis buffer (10 mM Tris-HCl pH 8.0, 5 mM EDTA, 10% SDS). An equal volume of phenol-chloroform was added, samples were mixed and centrifuged (15,000 g for 5 min, 4°C). The aqueous phase was saved and 100 mM NaCl and one volume isopropanol were added. After precipitation at -20°C for one hour, the samples were centrifuged (15,000 g, 20 min, 4°C) and the pellets were washed with 70% EtOH. The DNA was resuspended in 100 μl TE buffer. DNA concentrations were measured using the Qubit fluorometric instrument (ThermoFisher Scientific).

### Primer extension

Isolated DNA (1.8 μg) was incubated for 1 hour at 37°C with or without RNase H2 (NEB). Primer extension was performed with 2 U Taq DNA polymerase (NEB) in 1X ThermoPol buffer, 200 μM dNTPs and 1.5 pmol labeled primer. The primers were 5´-end labeled with PNK (NEB) and γ-[^32^P]ATP and were corresponding to L-strand positions 8-29 (GGT CTA TCA CCC TAT TAA CCA C) and 16,331-16,351 (CAC ACA TCA ACT GCA ACT CCA). The primer extension reaction was performed with 5 minutes at 95°C, 30 seconds at 95°C, 30 seconds at 58°C, 45 seconds at 72°C and 5 minutes at 72°C with step 2-4 repeated in 20 cycles. The reactions were stopped and ethanol precipitated as for replication initiation reactions. The sequencing ladders were prepared with USB Sequenase Version 2.0 (Affymetrix) according to the manufacturers protocol. The primer extension experiment was performed multiple times with similar results and the figure shows a representative gel image for that experiment.

### 5′-End-Seq and HydEn-Seq

Free 5′-ends of mtDNA from fibroblast cells were mapped by 5′-End-seq or HydEn-seq essentially as previously described [43,57]. In brief, 1 μg DNA was treated with 0.3 M KCl or 0.3 M KOH at 55°C for 2 hours. Samples were then phosphorylated with 3′-phosphatase-minus T4 polynucleotide kinase (New England BioLabs) for ligation to oligonucleotide ARC140. After an adaptor was annealed (ARC76–ARC77), T7 DNA polymerase (New England BioLabs) was used to synthesize the second strand. Purified libraries were then sequenced using an Illumina NextSeq500 instrument. Reads were trimmed for quality and adapter sequence with cutadapt (-m 15 –q 10match-reas-wildcards). Pairs with one or both reads shorter than 15 nts were discarded. Mate 1 of the remaining pairs was aligned to an index containing the sequence of all oligos used in the preparation of these libraries with bowtie sing bowtie 0.12.8 (-m1 -v2), and all pairs with successful alignments were discarded. Pairs passing this filter were subsequently aligned to the hg38 *H*. *sapiens* reference mitochondrial genome where the mitochondrial genome was cleaved at position 4,000 and OriH region was religated (-m1 -v2 -X10000 --best). Single-end alignments were then performed using mate 1 of all unaligned pairs (-m1 -v2). The count of 5′-ends of all unique paired-end and single-end alignments were determined and these counts were converted to bedGraph format for visualization. Sequencing data have been deposited in the Gene Expression Omnibus under accession number GSE103612.

## Supporting information

S1 Fig Mapping of replication initiation sites. To conclusively demonstrate that replication was indeed initiated in the CSBII-CSBIII region in our plasmid templates, we precisely mapped the RNA-DNA transition points. To do this, we created mutant templates where the sites for terminating ddCTP incorporation in the CSBII-CSBIII region were changed. The unmodified template contained three different reading frames, which allowed the formation of replication products no longer than 19 nts (frame 1), 18 nts (frame 2) or 41 nts (frame 3) (S1A Fig). To map the individual replication products to the individual reading frames we either truncated the frames by ~10 nts by inserting an additional cytosine (+C), or increased the length of the native frame by inserting one additional nucleotide just before the stopping cytosine (+1). In this way, we could map individual replication products to individual reading frames. Once we had defined the 3′-ends of individual replication products in this manner, we could, based on the length of the replication products, easily deduce the position of their respective 5′-ends (S1A and S1B Fig). We performed replication initiation reactions using these mutant templates (S1C Fig) and the results showed that nearly all replication products observed originated from the CSBIII and CSBII regions. The locations of major replication initiation sites are indicated with asterisks in S1A Fig. A. Location of three possible replication product frames in the CSBII-III region with at least 15 nts between adjacent cytosines. The location of the RNA to DNA transitions mapped in panel C are indicated with asterisks. B. To identify the replication products observed, we used mutant templates where we had truncated the frames by ~10 nts by inserting additional cytosines (+C) or increased the length of the native frame by inserting one additional nucleotide just before the stopping cytosine (+1). C. Replication initiation experiment with mutant templates described in B. The Frame 1 mutant templates appeared to alter the position of a couple of weak bands around 15 and 20 nts (compare lanes 1 to 2-3). When examining the Frame 2 mutants, most of the stronger products shorter than 20 nts clearly disappeared in the +C template and increased by one nucleotide in the +1 template (compare lanes 1 to 4-5). As for the Frame 3 mutant templates, all additional products, i.e. those longer than 20 nts, disappeared in the +C template and increased by one nucleotide in the +1 template (compare lanes 1 to 6-7). The correspondence between bands and frames is indicated in the WT lane cutout.
(TIF)

S2 Fig Replication initiation with RNase H1 mutants. Replication initiation as described in Fig 2A with 40 nM mtSSB and 2 nM RNase H1 WT or mutants as indicated.
(TIF)

S1 Table Description of templates used for transcription and replication experiments. (DOCX)
