# Supplementary figures and images for: RNase H1 directs origin-specific initiation of DNA replication in human mitochondria
Source: PLoS Genet. 2019 Jan 3;15(1):e1007781. doi: 10.1371/journal.pgen.1007781 (PMC6317783; doi:10.1371/journal.pgen.1007781)

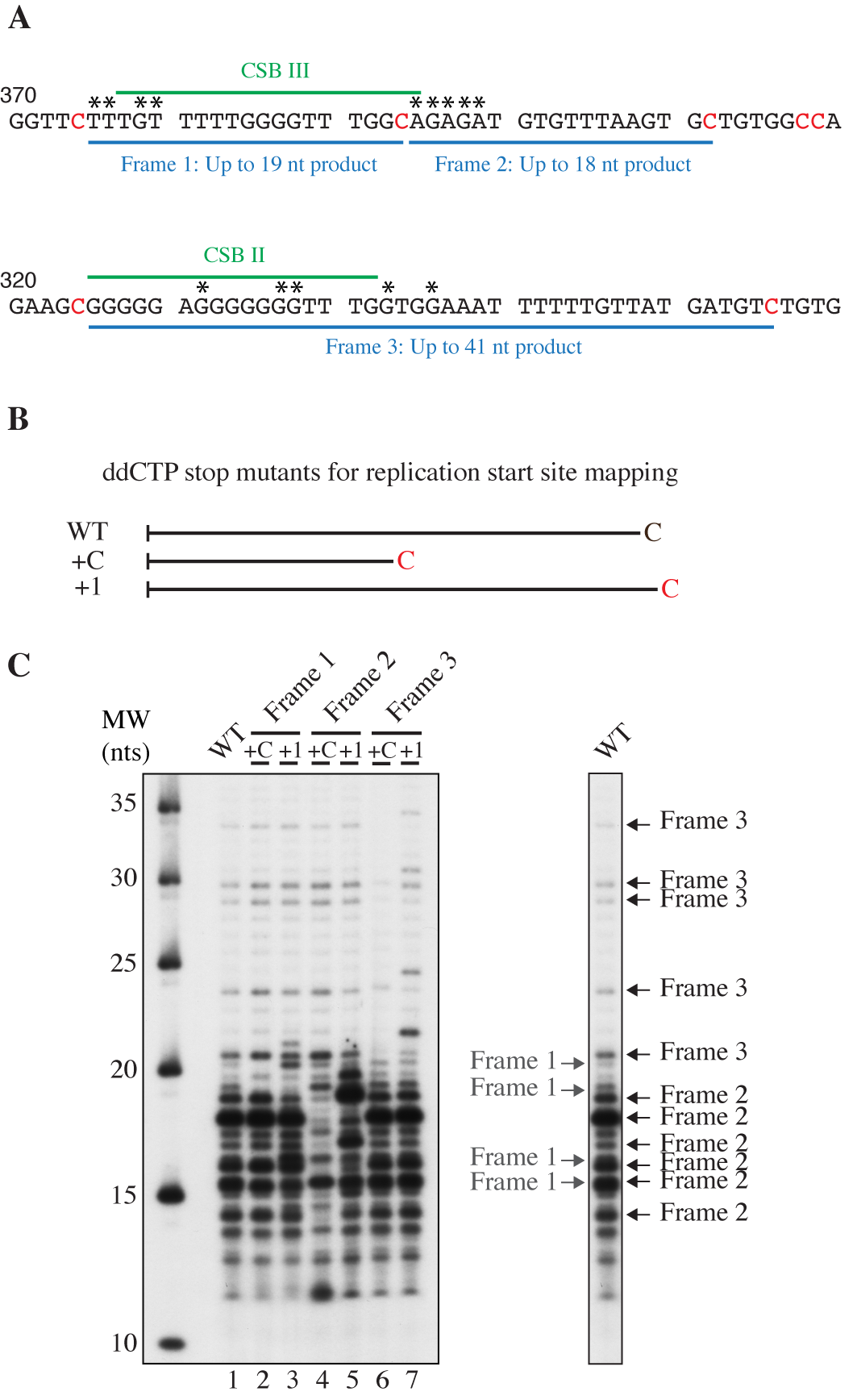

Supplement: S1 Fig — To conclusively demonstrate that replication was indeed initiated in the CSBII-CSBIII region in our plasmid templates, we precisely mapped the RNA-DNA transition points. To do this, we created mutant templates where the sites for terminating ddCTP incorporation in the CSBII-CSBIII region were changed. The unmodified template contained three different reading frames, which allowed the formation of replication products no longer than 19 nts (frame 1), 18 nts (frame 2) or 41 nts (frame 3) (S1A Fig). To map the individual replication products to the individual reading frames we either truncated the frames by ~10 nts by inserting an additional cytosine (+C), or increased the length of the native frame by inserting one additional nucleotide just before the stopping cytosine (+1). In this way, we could map individual replication products to individual reading frames. Once we had defined the 3′-ends of individual replication products in this manner, we could, based on the length of the replication products, easily deduce the position of their respective 5′-ends (S1A and S1B Fig). We performed replication initiation reactions using these mutant templates (S1C Fig) and the results showed that nearly all replication products observed originated from the CSBIII and CSBII regions. The locations of major replication initiation sites are indicated with asterisks in S1A Fig. A. Location of three possible replication product frames in the CSBII-III region with at least 15 nts between adjacent cytosines. The location of the RNA to DNA transitions mapped in panel C are indicated with asterisks. B. To identify the replication products observed, we used mutant templates where we had truncated the frames by ~10 nts by inserting additional cytosines (+C) or increased the length of the native frame by inserting one additional nucleotide just before the stopping cytosine (+1). C. Replication initiation experiment with mutant templates described in B. The Frame 1 mutant templates appeared to [file pgen.1007781.s001.tif]

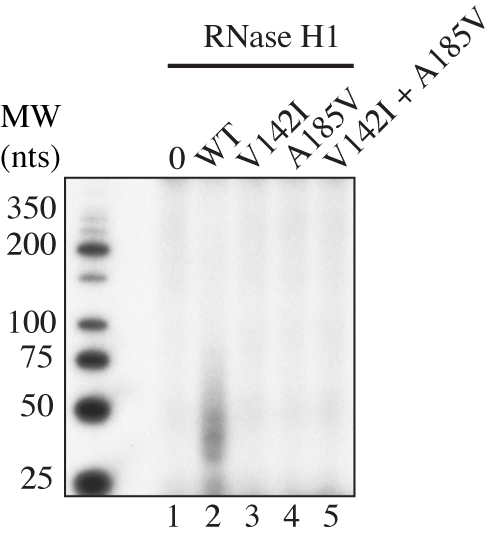

Supplement: S2 Fig — Replication initiation as described in Fig 2A with 40 nM mtSSB and 2 nM RNase H1 WT or mutants as indicated. (TIF) [file pgen.1007781.s002.tif]
