# Supplementary material for: RNase H1 directs origin-specific initiation of DNA replication in human mitochondria
Source: PLoS Genet. 2019 Jan 3;15(1):e1007781. doi: 10.1371/journal.pgen.1007781 (PMC6317783; doi:10.1371/journal.pgen.1007781)
Supplement: S1 Table — (DOCX) [file pgen.1007781.s003.docx]

| **Template** | **Derived from** | **Description** |
| --- | --- | --- |
| pUC-LSP | mtDNA+pUC18 | 1-477 cloned between BamHI and HindIII in pUC18 |
| pUC-HSP | mtDNA+pUC18 | 499-742 cloned between BamHI and HindIII in pUC18 |
| CSB2 mutant | LSP-pUC18 | G to A, A to G, C to T and T to C mutations of 296-315 |
| CSB3 mutant | LSP-pUC18 | G to A mutations of 353-356 and 348-349 |
| Upstream CSBIII (Frame 1) - | LSP-pUC18 | C insertion at position 357 (creating a new ddCTP termination site) |
| Upstream CSBIII (Frame 1) +1 | LSP-pUC18 | T insertion prior to the terminating C at 347 |
| Downstream CSBIII (Frame 2) - | LSP-pUC18 | C insertion at position 338 (creating a new ddCTP termination site) |
| Downstream CSBIII (Frame 2) +1 | LSP-pUC18 | T insertion prior to the terminating C at 329 |
| Upstream CSBII (Frame 3) - | LSP-pUC18 | C insertion at position 286 (creating a new ddCTP termination site) |
| Upstream CSBII (Frame 3) +1 | LSP-pUC18 | T insertion prior to the terminating C at 275 |
